# II Brazilian Society of Rheumatology consensus for lupus nephritis diagnosis and treatment

**DOI:** 10.1186/s42358-024-00386-8

**Published:** 2024-06-18

**Authors:** Edgard Torres dos Reis-Neto, Luciana Parente Costa Seguro, Emília Inoue Sato, Eduardo Ferreira Borba, Evandro Mendes Klumb, Lilian Tereza Lavras Costallat, Marta Maria das Chagas Medeiros, Eloisa Bonfá, Nafice Costa Araújo, Simone Appenzeller, Ana Carolina de Oliveira e Silva Montandon, Emily Figueiredo Neves Yuki, Roberto Cordeiro de Andrade Teixeira, Rosa Weiss Telles, Danielle Christinne Soares do Egypto, Francinne Machado Ribeiro, Andrese Aline Gasparin, Antonio Silaide de Araujo Junior, Cláudia Lopes Santoro Neiva, Debora Cerqueira Calderaro, Odirlei Andre Monticielo

**Affiliations:** 1 Division of Rheumatology, Department of Medicine, Escola Paulista de Medicina, Universidade Federal de São Paulo (EPM/Unifesp), Otonis Street, 863, 2 Floor, Vila Clementino, São Paulo, SP 04025-002 Brazil; 2 Division of Rheumatology, Hospital das Clínicas, Faculdade de Medicina da Universidade de São Paulo (FMUSP), São Paulo, Brazil; 3 Department of Rheumatology, Hospital Universitário Pedro Ernesto, Universidade do Estado do Rio de Janeiro, Rio de Janeiro, Brazil; 4 Division of Rheumatology, Department of Orthopedics, Rheumatology and Traumatology, Universidade Estadual de Campinas (Unicamp), Campinas, Brazil; 5 Division of Rheumatology, Universidade Federal do Ceará (UFC), Fortaleza, Brazil; 6 Division of Rheumatology, Hospital do Servidor Público Estadual de São Paulo - Instituto de Assistência Médica ao Servidor Público Estadual de São Paulo, São Paulo, Brazil; 7 Division of Rheumatology, Hospital das Clínicas da Universidade Federal de Goiás, Goiânia, Brazil; 8 Division of Rheumatology, Universidade Estadual de Ciências da Saúde de Alagoas, Maceió, Brazil; 9 Division of Rheumatology, Faculdade de Medicina da Universidade Federal de Minas Gerais (UFMG), Belo Horizonte, Brazil; 10 Division of Rheumatology, Department of Internal Medicine, Universidade Federal da Paraíba (UFPB), João Pessoa, Brazil; 11 Division of Rheumatology, Department of Internal Medicine, Hospital de Clínicas de Porto Alegre, Universidade Federal Do Rio Grande Do Sul, Porto Alegre, Brazil; 12 Division of Rheumatology, Santa Casa de Misericórdia de Belo Horizonte, Belo Horizonte, Brazil

## Abstract

**Objective:**

To develop the second evidence-based Brazilian Society of Rheumatology consensus for diagnosis and treatment of lupus nephritis (LN).

**Methods:**

Two methodologists and 20 rheumatologists from Lupus Comittee of Brazilian Society of Rheumatology participate in the development of this guideline. Fourteen PICO questions were defined and a systematic review was performed. Eligible randomized controlled trials were analyzed regarding complete renal remission, partial renal remission, serum creatinine, proteinuria, serum creatinine doubling, progression to end-stage renal disease, renal relapse, and severe adverse events (infections and mortality). The Grading of Recommendations Assessment, Development and Evaluation (GRADE) approach was used to develop these recommendations. Recommendations required ≥82% of agreement among the voting members and were classified as strongly in favor, weakly in favor, conditional, weakly against or strongly against a particular intervention. Other aspects of LN management (diagnosis, general principles of treatment, treatment of comorbidities and refractory cases) were evaluated through literature review and expert opinion.

**Results:**

All SLE patients should undergo creatinine and urinalysis tests to assess renal involvement. Kidney biopsy is considered the gold standard for diagnosing LN but, if it is not available or there is a contraindication to the procedure, therapeutic decisions should be based on clinical and laboratory parameters. Fourteen recommendations were developed. Target Renal response (TRR) was defined as improvement or maintenance of renal function (±10% at baseline of treatment) combined with a decrease in 24-h proteinuria or 24-h UPCR of 25% at 3 months, a decrease of 50% at 6 months, and proteinuria < 0.8 g/24 h at 12 months. Hydroxychloroquine should be prescribed to all SLE patients, except in cases of contraindication. Glucocorticoids should be used at the lowest dose and for the minimal necessary period. In class III or IV (±V), mycophenolate (MMF), cyclophosphamide, MMF plus tacrolimus (TAC), MMF plus belimumab or TAC can be used as induction therapy. For maintenance therapy, MMF or azathioprine (AZA) are the first choice and TAC or cyclosporin or leflunomide can be used in patients who cannot use MMF or AZA. Rituximab can be prescribed in cases of refractory disease. In cases of failure in achieving TRR, it is important to assess adherence, immunosuppressant dosage, adjuvant therapy, comorbidities, and consider biopsy/rebiopsy.

**Conclusion:**

This consensus provides evidence-based data to guide LN diagnosis and treatment, supporting the development of public and supplementary health policies in Brazil.

**Supplementary Information:**

The online version contains supplementary material available at 10.1186/s42358-024-00386-8.

## Introduction

Systemic lupus erythematosus (SLE) is a heterogeneous and pleomorphic systemic autoimmune disease characterized by periods of activity and remission with high rates of organ damage and morbimortality [1]. The incidence of SLE in the city of Natal/Brazil was 8.7 cases per 100,000 inhabitants per year in 2000 [2], and it is currently estimated that there are 150,000 to 300,000 people with SLE in the country [3].

Lupus nephritis (LN) occurs in up to 50% of adults with SLE and 80% of juvenile-onset SLE patients, and up to 30% progress to end-stage chronic kidney disease (CKD) in 15 years [3], with impaired quality of life and socioeconomic impact. Therefore, the early recognition of LN is very important to initiate appropriate treatment that could modify the course of the disease and improve its prognosis.

The last consensus of the Brazilian Society of Rheumatology for LN treatment was published in 2015 [4]. Since then, specific targets, new treatment options and novel biomarkers to help diagnosis and monitoring SLE have been described. In view of these new available data, associated with the high frequency of SLE in Brazil and the morbimortality of the disease, there is an urgent need to update the Consensus for diagnosis and treatment of LN, which may support decision in clinical practice as well as the development of public and supplementary health policies in Brazil.

## Methods

This consensus, supported by the Brazilian Society of Rheumatology, was developed by a team of two methodologists and 20 rheumatologists with experience in SLE, members of the SLE Committee of the SBR, who defined 14 PICO questions (*population, intervention, comparator, outcome*) on different aspects of LN treatment (Supplementary Material 1). This study was conducted using a systematic review model according to the international recommendations of the Preferred Reporting Items for Systematic Reviews and Meta-Analyses (PRISMA). Eligible randomized controlled trials (RCTs) were included and analyzed: complete renal remission, partial renal remission, serum creatinine, proteinuria, serum creatinine doubling, progression to end-stage renal disease (CKD), renal relapse, and severe adverse events (infections and mortality). The following databases were used for research (supplementary material 2): the Cochrane Central Register of Controlled Trials (CENTRAL) in the Cochrane Library (2021, Edition 7), MEDLINE via PubMed (1966 to July 13, 2021), Embase via Elsevier (1974 to July 13, 2021), and Lilacs via the Virtual Health Library (VHL) Regional Portal (1982 to July 13, 2021).

**Fig. 1:**
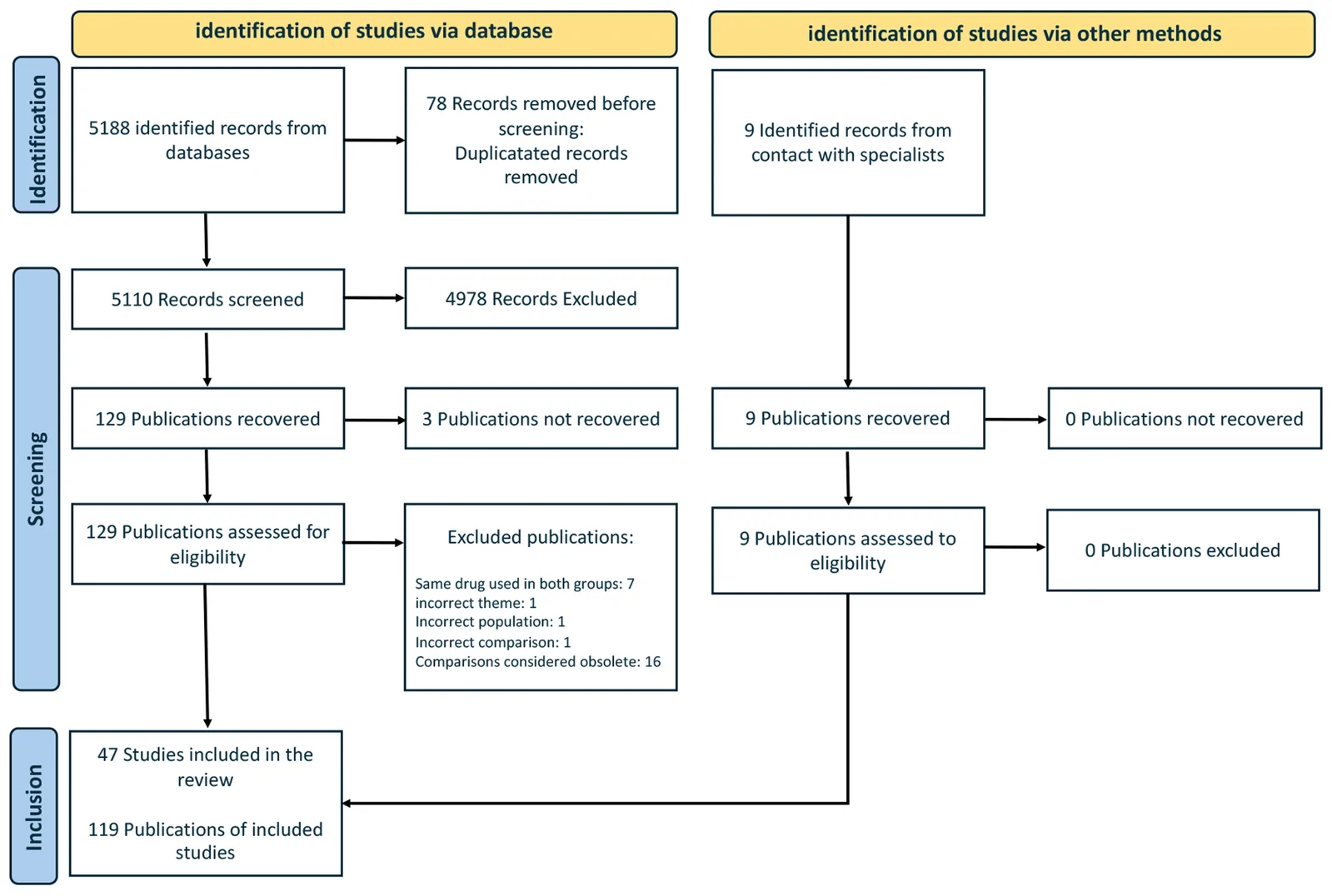
Literature search flowchart

The triage process was performed using Rayyan software. Two authors (ETRN and LPCS) independently selected the titles and abstracts and identified studies that met the eligibility criteria. For the studies included in the first phase, the full texts were retrieved, and eligibility for definitive inclusion was assessed. In cases of discrepancy, a third reviewer (VTC) was consulted. Information regarding the selection stage is described in the PRISMA flowchart (Fig. 1). Extraction and management of data were independently performed by two methodologists (VTC and NCJ), who assessed the risk of bias of each included study using version 2 of the Cochrane risk of bias tool version 2 (RoB2) according to the recommendations of the Cochrane Handbook for Systematic Reviews of Interventions, version 6.0.

Tables of the main findings for all outcomes were created. GRADEpro Guideline Development Tool (GDT) software (GRADEpro GDT, McMaster University and Evidence Prime Inc., McMaster University, Hamilton, Ontario, Canada) was used to analyze the overall certainty of the evidence, and each outcome was categorized into four levels of certainty: high, moderate, low, and very low [5]. The recommendations were prepared according to the Grading of Recommendations Assessment, Development, and Evaluation (GRADE) Working Group guidelines, especially regarding problem priority, balance between benefits and harms, patients’ values and preferences, costs, health equity, acceptability, and feasibility. Seventeen rheumatologists evaluated all the evidence and voted on the recommendations for each PICO question. Recommendations required ≥82% (14/17) agreement among the voting members. The recommendations for each PICO question were classified as follows: strongly in favor, weakly in favor, conditional, weakly against, and strongly against a particular intervention.

Other aspects of LN management (diagnosis, general principles of treatment, treatment of comorbidities and refractory cases) were evaluated through literature review and expert opinion.

## Diagnoses of lupus nephritis

All patients diagnosed with SLE, even if asymptomatic, should undergo creatinine and urinalysis tests to assess renal involvement. The frequency must be personalized in each case, from 1 to 3 months in the induction therapy to 3 to 6 months in maintenance therapy or asymptomatic patients [6, 7]. LN is defined by the presence of persistent proteinuria (>500 mg in 24 h or urinary protein/creatinine ratio (UPCR) > 0.5) and/or active urinary sediment (dysmorphic hematuria or presence of hemoglobin, red blood cells, granular, tubular, or mixed casts) in the absence of infection or another explanation, or by renal biopsy demonstrating immune-mediated glomerulonephritis [8]. However, lower levels of proteinuria may be present in patients with active proliferative nephritis, which has been called “silent nephritis” [9, 10].

According to the analysis of this consensus, 24-h proteinuria and spot UPCR demonstrated a strong correlation [r = 0.82 (0.76–0.83)] (supplementary material 3). However, there was high heterogeneity and low agreement between studies, especially when 24-h proteinuria was less than 500 mg or between 500 mg and 1 g [11–13]. Thus, although spot UPCR is a great test for screening and monitoring patients with LN, we recommend that 24-h proteinuria or 24-h UPCR should be used as the most accurate measure for decisions in clinical practice, including changes in clinical scenarios or in immunosuppressive therapy.

Anti-dsDNA and anti-C1q antibodies are useful for the diagnosis and monitoring of LN activity, especially in proliferative classes. Although the anti-C1q antibody test is not widely available in clinical practice in Brazil, if both are present, the positive predictive value is 67% for LN activity [14, 15]. The presence of anti-C1q, anti-dsDNA, and complement consumption increases the risk of LN (OR 14.9; 95% CI 5.8–38.4) [16]. On the other hand, it is important to reinforce that it is not necessary to treat SLE patients with anti-dsDNA antibodies or complement consumption without clinical disease manifestations [17, 18]. Anti-nucleosome antibodies are strongly correlated with anti-dsDNA antibodies and appear earlier in active LN [19]. The presence of anti-P-ribosomal antibodies seems to be related to class V, conferring a better prognosis, especially in the absence of anti-dsDNA antibodies [20, 21]. Anti-neutrophil cytoplasmic antibodies (ANCA), especially the p-ANCA pattern associated with anti-myeloperoxidase (MPO) antibodies, can be detected in LN, particularly in class IV with higher creatinine level and worse prognosis [22–24].

Kidney biopsy is considered the gold standard for diagnosing LN, establishing histological class (glomerulonephritis classes I to VI), evaluating parameters of activity (graded from 0–24) and chronicity (graded from 1–12) and guiding treatment according to previous published guidelines (Table 1) [25–27]. Tubulointerstitial and vascular involvements should also be analyzed to determine patient prognosis and support differential diagnosis [28]. It is also useful to identify other pathologies, such as hypertensive or diabetes nephropathy and thrombotic microangiopathy (TMA), which may have implications in treatment decisions and prognosis [29–31]. Biopsy should be performed when there is suspicion of renal involvement in SLE, including at least one of the following [32, 33]:

- 24-h proteinuria ≥ 500 mg or UPCR ≥ 0.5
- Abnormal renal function (increase in serum creatinine >30% or decrease in glomerular filtration rate, GFR) of unknown etiology
- Glomerular hematuria with proteinuria <0.5 g/24 h
- Differential diagnosis with other conditions, such as hypertension, diabetes mellitus, TMA, podocytopathy, tubulointerstitial lesions, collapsing glomerulopathy, infections, and others.

**Table 1:** Classification of lupus nephritis and indices of activity and chronicity on kidney biopsy

**Class I**	**Minimum Mesangial**	
**Class II**	**Proliferative Mesangial**	
**Class III**	**Focal**	
	Focal active or inactive, segmental or global, endo or extra capillary glomerulonephritis involving <50% of all glomeruli	
**Class IV**	**Diffuse**	
	Diffuse active or inactive, segmental or global, endo- or extra capillary glomerulonephritis involving ≥50% of all glomeruli; diffuse segmental (IV-S) in which ≥50% of the involved glomeruli have segmental lesions (involving less than half of the tuft); or global diffuse (IV-G) in which ≥50% of the involved glomeruli have global lesions (involving more than half the tuft)	
**Class V**	**Membranous**	
	May occur in combination with classes III or IV	
**Class VI**	**Advanced sclerosis**	
	Global glomerular sclerosis in ≥90% without residual activity	
**Activity index (0–24)**	**Chronicity index (0–12)**	**Score**
• Endocapillary hypercellularity	• Total glomerular sclerosis	• 0: Absent
• Neutrophils and/or karyorrhexis	• Fibrous crescents	• 1: <25%
• Hyaline deposits	• Interstitial fibrosis	• 2: 25–50%
• Fibrinoid necrosis (x2)	• Tubular atrophy	• 3: >50%
• Cellular/fibrocellular crescents (x2)		
• Interstitial inflammation		

Adapted from Weening et al. [25], Bajema et al. [26], and Austin et al. [27]

On the other hand, the delay in starting immunosuppressive treatment (especially in suspected cases of rapidly progressive glomerulonephritis) is associated with worse short- and long-term renal prognoses. Therefore, if kidney biopsy is not available or there is a contraindication to the procedure, therapeutic decisions should be based on clinical and laboratory parameters [34]. Classes I and II represent mesangial involvement that usually manifests with few clinical symptoms and laboratory abnormalities, and there may be mild proteinuria (usually <1 g/24 h) and dysmorphic hematuria. Classes III and IV usually present proteinuria above 500 mg/24 h, dysmorphic hematuria, and/or the presence of red blood cell casts. Hypertension, loss of renal function and rapidly progressive glomerulonephritis can occur in severe cases. Pure class V comprises only 10% to 20% of LNs and usually presents with nephrotic syndrome without leukocyturia or hematuria; also, class V can be associated with class III or IV [III or IV (±V)] [35, 36]. A Brazilian study developed an instrument to differentiate classes III or IV (±V) from class V based on clinical and laboratory parameters (https://ppg.unifesp.br/reumato/comunicados/lupus-nephritis) [36], which can help clinical decision if kidney biopsy is not available.

Kidney biopsy should be repeated in cases of refractory disease (persistent proteinuria after one year and/or worsening of serum creatinine) or LN relapse [29–31].

**Practical issues for lupus care:** although biopsy remains the gold standard for LN diagnosis, accessibility is limited in Brazil. Therefore, the use of clinical and laboratorial parameters remains the mainstay for diagnosis in most regions of our country. An instrument was recently published in order to differentiate LN classes [36].

## Treatment target: Target Renal Response (TRR)

Assessment of proteinuria is essential in the management of LN since early reduction in proteinuria level is a predictor of renal response. A 1-year proteinuria level <0.7–0.9 g/24 h is the best predictor of long-term renal outcome, assessed by important LN cohorts (Euro-Lupus and MAINTAIN nephritis trials) and by two Brazilian studies including patients with severe disease in real life situation [37–41]. Other predictive parameters of favorable renal outcome are a reduction in proteinuria of 25% at week 8 [42, 43], a significant decrease in proteinuria at week 12 [39, 44], a reduction ≥50% from baseline at 6 months [44, 45], and proteinuria ≤1 g at 6 months of treatment [46].

Thus, the panelists considered the improvement or maintenance of renal function (±10% at baseline of treatment) combined with a decrease in 24-h proteinuria or 24-h UPCR of 25% at 3 months, a decrease of 50% at 6 months, and proteinuria <0.8 g/24 h at 12 months as the targets of response to treatment; these targets were called the Target Renal Response (TRR) (94.1% agreement). Patients with nephrotic proteinuria at baseline may require an additional 6–12 months to achieve TRR. In these patients, immediate changes to therapy are not necessary if proteinuria improves [47]. On the other hand, if there is no clinical or laboratory improvement or worsening within 3 months, a change in therapy should be considered.

**Fig. 2:**
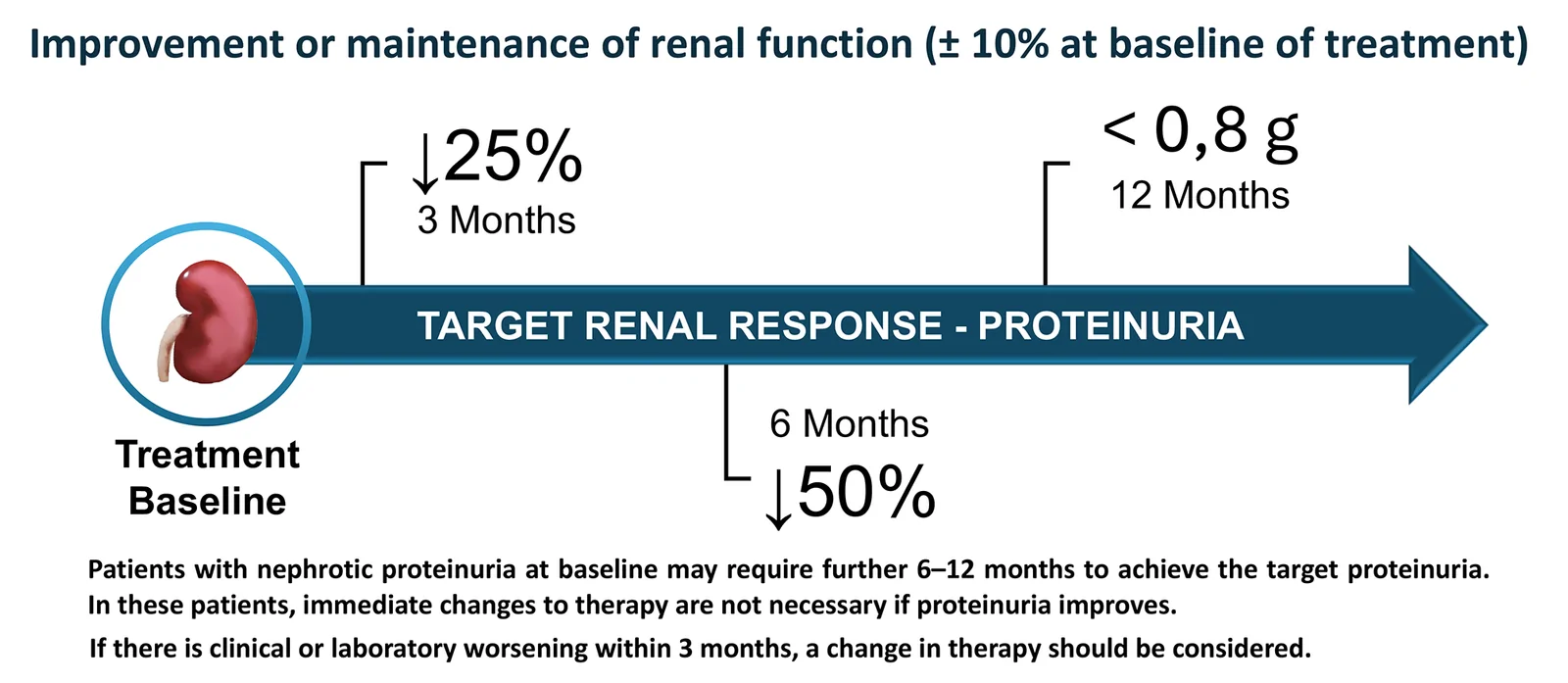
Treatment target for lupus nephritis: Target Renal Response (TRR)

**Practical issues for lupus care:** assessment of Target Renal Response (corresponding to a reduction in proteinuria levels at 3 months, 6 months and 12 months after treatment, with preserved renal function compared to baseline), is an easy and effective way to evaluate renal response. The target of proteinuria <0.8 g/day at 12 months was defined according to data from Brazilian patients [40, 41] (Fig. 2).

## Duration of immunosuppressive treatment

Induction immunosuppressive treatment (initial therapy) should last 3 to 6 months and should be followed by maintenance treatment (sequential treatment), lasting at least 3 to 5 years in those who achieve TRR. Immunosuppressive treatment for 4 to 5 years was associated with a lower risk of renal relapse than treatment for 2 to 3 years [48]. The suspension should be gradual and individualized, and should be carried out under medical supervision, taking into account renal response, number of previous renal relapses, duration of the remission period, presence of renal damage, extrarenal activity, patient preferences and may be guided by biopsy [49].

There is a discussion on the role of rebiopsy after induction (initial) and/or maintenance (sequential) therapy to identify patients who need to prolong or intensify therapy (patients who persist with histological activity), as well as candidates to discontinue immunosuppressive treatment (patients with complete histological response or activity index ≤2) [50–52].

**Practical issues for lupus care:** maintenance (sequential) treatment should last at least 3 to 5 years. Patients with incomplete response, with multiple previous relapses or with renal damage might need longer periods of immunosuppressive treatment.

## Definition of refractory lupus nephritis

This topic is a subject of debate and still lacks consensus, given the wide variety of criteria used by different authors [53]. Refractoriness can be understood as the impossibility of achieving remission of the renal inflammatory process despite appropriate treatment [54, 55].

According to this consensus, refractory LN was defined when TRR was not achieved by at least two regimens of induction (initial) therapy or when there were contraindications to other proposed treatments, confirming that there was adherence to treatment.

The persistence of proteinuria and renal dysfunction does not always indicate persistent immune-mediated activity. Proteinuria may result from a lack of adherence to treatment, inadequate control of comorbidities (hypertension, diabetes, infections), drug nephrotoxicity, the presence of other concomitant renal diseases (e.g., TMA, other glomerulopathies), genetic factors (e.g., APOL1 variants, pharmacogenetic resistance to immunosuppressive medications) or LN with a predominance of irreversible lesions (damage). Patients with poor adherence to treatment have more LN relapse and are more susceptible to refractory disease [56].

## Factors associated with worse renal prognosis in LN

- Patient characteristics: male sex, juvenile-onset lupus, increased serum creatinine or proteinuria >4 g at diagnosis, frequent relapses, incomplete remission, neuropsychiatric lupus, and thrombocytopenia at diagnosis.
- Serological characteristics: positive antiphospholipid antibodies (aPLs) or antiphospholipid syndrome (APS), high-titer anti-dsDNA, anti-C1q, and persistent complement consumption.
- Histological features: crescents, TMA or tubulointerstitial damage (interstitial fibrosis, tubular atrophy and interstitial inflammation) [33].

## Treatment of class III or IV lupus nephritis with or without class V [class III or IV (±V)]

**Fig. 3:**
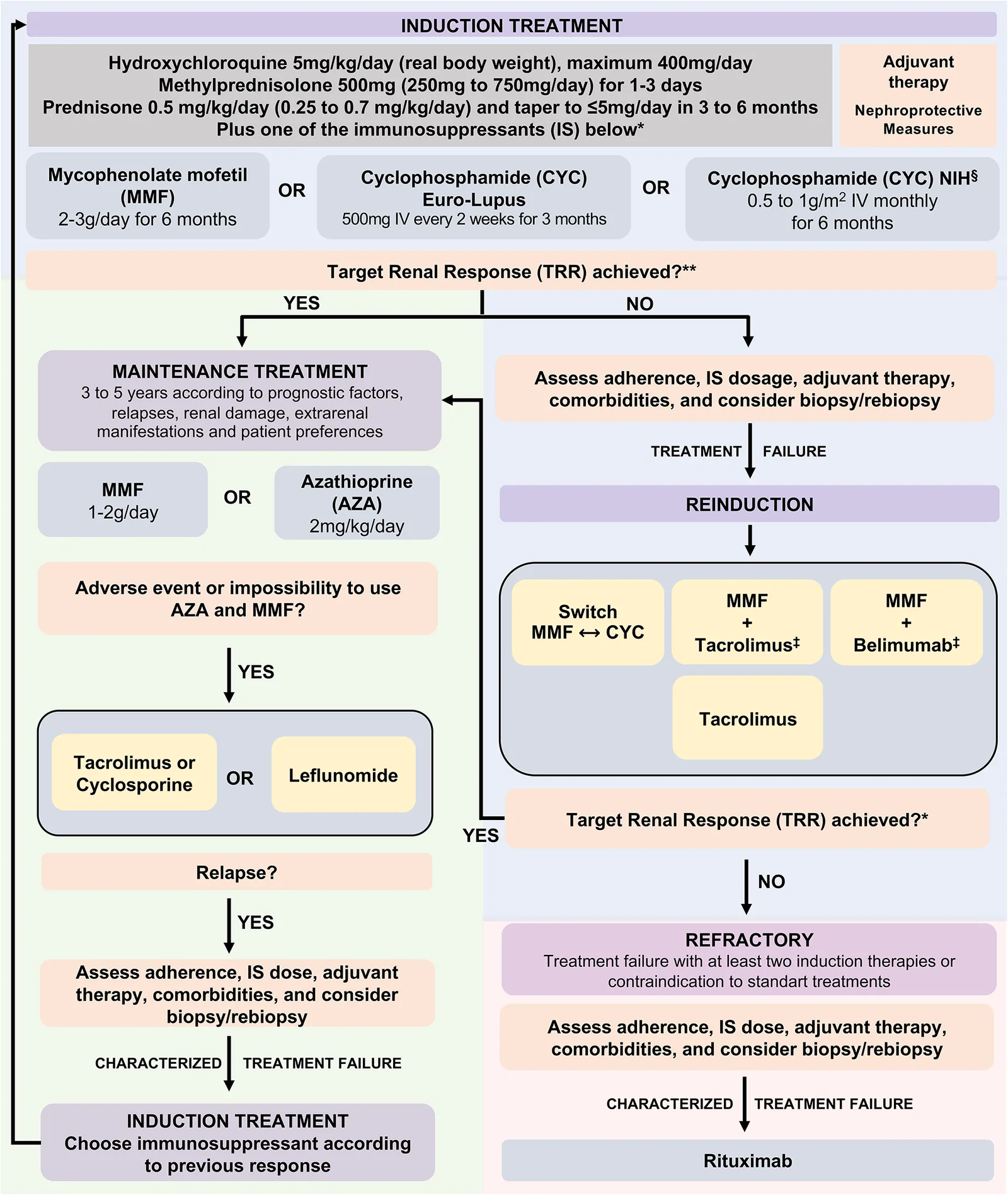
Treatment of class III or IV LNs (±V). *Factors to be considered when choosing immunosuppressants: severity, availability, adherence, infusion clinic availability, gastrointestinal tolerance, cumulative dose of CYC, age/fertility, desire for pregnancy. **Target Renal Response (TRR): reduction in proteinuria by 25% at 3 months, 50% at 6 months, and proteinuria < 0.8 g at 1 year associated with maintenance or improvement (±10% baseline) in renal function. Nephrotic proteinuria at baseline may require another 6–12 months to achieve TRR and, in such cases, immediate therapy changes are not necessary if proteinuria is improving. If clinical or laboratory results worse within 3 months, therapy changes should be considered. ^**§**^Severe Disease, Poor prognostic factors, Impossibility to MMF or CYF Euro-Lupus. ^**‡**^In case of TRR achieved, Mycophenolate + Belimumab or Mycophenolate + Tacrolimus can be used as maintenance therapy for up to 3 years

Recommendations, as well as their strength and certainty of evidence, are shown in Table 2 and the treatment flowchart is shown in Fig. 3. For immunosuppressant choice, the following factors should be considered: severity, adherence, availability/access to medication and infusion centers, pregnancy or lactation, risk of infertility, costs, and patient preference (Table 3).

**Table 2:** Principles and recommendations for the treatment of class III or IV (±V) lupus nephritis

**Recommendation**	**Level of agreement**
1. HCQ should be prescribed to all SLE patients, except if contraindicated.	100%
2. Glucocorticoids should be used at the lowest dose and for the minimal necessary period.	100%
**Induction therapy**	
3. Initial induction therapy involves the use of MMF or intravenous CYC	94.1%
4. The combination of MMF and TAC can be used as induction therapy, particularly if there is lack of response or impossibility to use CYC or higher doses of MMF (induction dose).	82.3%
5. The combination of BEL and MMF can be used as induction therapy according to specific characteristics of the patient	94.1%
6. TAC as immunosuppressant in monotherapy can be used as induction therapy if MMF, CYC, MMF + TAC or BEL + MMF cannot be used	100%
7. The combination of MMF and voclosporin may be considered for induction therapy after its approval by Brazilian regulatory agencies	94.1%
8. CsA as immunosuppressant in monotherapy is not recommended for induction therapy	82.3%
9. LFN as immunosuppressant in monotherapy is not recommended for induction therapy	88.9%
10. Monthly glucocorticoid pulse therapy is not recommended during induction therapy	88.2%
**Maintenance therapy**	
11. Both MMF or AZA can be used as maintenance therapy	82.3%
12. Calcineurin inhibitors (TAC or CsA) can be used as maintenance therapy in patients who cannot use MMF or AZA	94.1% (CsA)
	100% (TAC)
13. LFN can be used as maintenance therapy in patients who cannot use MMF or AZA	94.1%
14. CYC is not recommended for maintenance therapy	94.1%

*AZA* Azathioprine, *BEL* Belimumab, *CYC* Cyclophosphamide, *CsA* Cyclosporine, *LEF* Leflunomide, *MMF* Mycophenolate mofetil, *TAC* Tacrolimus

**Table 3:** Factors to be considered when choosing immunosuppressants in clinical practice

	MMF	CYC NIH	CYC Euro-Lupus	AZA	TAC	CsA	BEL	RTX
Favors adherence		X	X					
Easy access	X	X	X	X		X		
Need of infusion center		X	X				X	X
Severe cases or refractory		X						X
Compatible with pregnancy				X	X	X		
Compatible with lactation				X	X	X		
Risk of infertility		X*	X*					
High cost					X		X	X

*AZA* Azathioprine, *BEL* Belimumab, *CYC* Cyclophosphamide, *CsA* Cyclosporine, *MMF* Mycophenolate mofetil, *RTX* Rituximab, *TAC* Tacrolimus

*Risk related to CYC cumulative dose and patient age

### Question: Should hydroxychloroquine (HCQ) be prescribed to all SLE patients with LN?

**Recommendation 1: HCQ should be prescribed to all SLE patients, except if contraindicated. Agreement: 100%.**

The use of antimalarial drugs is associated with numerous beneficial effects in SLE patients, including a higher remission rate of LN; a reduction in thrombotic risk; improved lipid and glycemic profiles; lower risks of infection, hospitalization, and progression to metabolic syndrome; better MMF response; damage prevention; and longer survival [57]. Due to its safety profile, HCQ is preferred over chloroquine diphosphate (CDF).

The recommended dose of HCQ is 5 mg/kg/day of real body weight (maximum dose of 400 mg/day), and for DFQ is 2.3 mg/kg/day (maximum dose of 250 mg/day) [58]. The dose should be reduced by 50% in patients with GFR < 30 ml/min [29]. Studies evaluating blood levels of HCQ are useful both for assessing adherence and for monitoring adequate target levels [59–62]. Of note, in obese patients (BMI ≥ 30 kg/m^2^), dose of HCQ should not exceed 5 mg/kg/day of ideal body weight, since it has been demonstrated that even with the recommended maximum daily dose restriction (400 mg/day), these patients have very high HCQ blood levels [59].

Maculopathy caused by HCQ stands out as one of the most significant adverse events. The major risk factors for retinal toxicity are the use of HCQ and DFQ above the recommended doses, an extended usage exceeding 5 years, impaired renal function, concomitant use of tamoxifen, and previous macular or retinal disease. Ophthalmological evaluation should be performed at the initiation of HCQ therapy and subsequently on annual basis for patients with risk factors for retinal toxicity. Additionally, baseline and after 5 years (annually after this period) for those without risk factors [58]. More sensitive tests, such as spectral domain-optical coherence tomography (OCT-SD) and automated threshold visual field tests, are recommended for detecting early retinal toxicity [63].

**Practical issues for lupus care:** HCQ should be prescribed to all SLE patients, except if contraindicated. More sensitive tests, such as OCT-SD, are recommended for detecting early retinal toxicity. Therefore, avoiding excessive HCQ doses is important to prevent retinal toxicity. Obese patients (BMI ≥ 30 kg/m^2^) should use 5 mg/kg/day of ideal body weight (maximum 400 mg/day), as suggested by a recent Brazilian study [59].

### Question: How should glucocorticoids be used in induction and maintenance therapy?

**Recommendation 2: Glucocorticoids should be used at the lowest dose and for the minimal necessary period. Agreement: 100%.**

Glucocorticoids (GC) exert rapid effect on the inflammatory process, with immediate benefits in controlling disease activity [64]. However, they are associated with several adverse events and damage accrual [65]. GC are related to 58% of first year damage and 80% of late damage (after 15 years of disease) [65, 66]. Some protocols using lower doses of GC have shown similar efficacy with less adverse events [67, 68]. GC are recommended for induction (initial) treatment, using the lowest dose, for the minimal necessary period and associated with immunosuppressants.

The consensus suggests that intravenous (IV) pulse therapy with methylprednisolone should be given at a preferred dose of 500 mg/day (ranging from 250 to 750 mg/day) for 1–3 days, followed by oral prednisone 0.5 mg/kg/day (ranging from 0.25 to 0.7 mg/kg/day), with progressive dose reduction and a target of ≤5 mg/day in 3 to 6 months.

**Practical issues for lupus care:** Similarly to other international recommendations, this consensus strongly recommends that glucocorticoids should be used at the lowest dose and for the minimal necessary period, in order to prevent damage accrual.

## Induction therapy

### Question: Is there a preference for mycophenolate mofetil (MMF) or cyclophosphamide (CYC) as induction therapy for LN?

**Recommendation 3: Induction therapy should involve the use of either MMF or intravenous CYC. Strength of recommendation: conditional. Certainty of evidence: moderate. Agreement: 94.1%.**

In class III or IV LN (±V), induction treatment includes either MMF (2 to 3 g/day) or intravenous CYC at a dose of 500 mg every 2 weeks for 3 months [EuroLupus Nephritis Trial protocol (Euro-Lupus)]. National Institutes of Health (NIH) CYC protocol, with monthly doses of 0.5–1.0 g/m^2^ of body surface area for 6 months, involves higher CYC cumulative dose. The pooled analysis of data from RCTs comparing these strategies showed, with moderate certainty of evidence, similar complete and partial remission rates between the groups at the 24-week evaluation. There was also no significant difference between the groups in terms of serum creatinine or proteinuria, although there was certainly weak evidence for these outcomes. Likewise, there was no significant difference between treatments regarding important adverse events such as infections and mortality [44, 69–77]. In the Aspreva Trial, MMF and CYC had similar efficacy overall to short-term induction therapy for LN and more Black and Hispanic patients responded to MMF than IVC. However, as these factors are inter-related, it is difficult to draw firm conclusions about their importance [78]. In cases of gastrointestinal intolerance to MMF, mycophenolate sodium (MFS) may be administered at a dosage of 1.44 to 2.16 g/day [79].

The choice of medication should consider factors such as patient age, desire for pregnancy, risk of infertility or early menopause, previous cumulative dose of CYC, availability of an infusion center, patient adherence to medications, previous gastrointestinal intolerance to MMF/MFS, and clinical and histological parameters of severity. In patients of childbearing age, the risk of infertility associated with CYC must be clearly shared with the patient, particularly when administered at high doses.

The Euro-Lupus protocol presents a lower cumulative CYC dose, and it has similar efficacy to that of the NIH regimen after a 10-year follow-up period [44, 77], including in patients outside European continent [70]. Euro-Lupus CYC Pivotal studies excluded patients with crescentic glomerulonephritis or a GFR < 25–30 mL/min [70–72, 74–77]. Post hoc analysis of the ASPREVA Lupus Management Study revealed no difference in response to treatment with MMF or the CYC NIH in patients with a GFR < 30 mL/min. However, there were few patients in each group, and the pivotal study was not designed for this purpose [80]. Therefore, considering the efficacy data and the increased risk of infertility and early menopause with higher doses of CYC, the CYC NIH regimen should be reserved for patients with poor prognostic factors, such as GFR < 30 mL/min or kidney biopsy with cellular crescents, fibrinoid necrosis, or severe tubulointerstitial nephritis in ≥50% of glomeruli, as well as for patients treated in centers with structural limitations to provide infusions or clinical visits more frequently (every 2 weeks).

**Practical issues for lupus care**: either MMF or CYC can be used as first line immunosuppressive drugs for induction LN therapy. MMF was recently incorporated into the treatment of LN by the Public Health System in Brazil, which simplifies patients’ access to medication. IV CYC is preferred for non-adherent patients to oral medication although it requires an infusion center. CYC NIH should be reserved for patients with more severe forms of LN due to higher CYC cumulative doses and adverse events, including infertility.

### Question: Can the combination of MMF and tacrolimus (TAC) be used as induction therapy for LN?

**Recommendation 4: The combination of MMF and TAC can be used in induction therapy, particularly if there is a lack of response or impossibility to use CYC or higher doses of MMF (induction dose). Strength of recommendation: weakly in favor. Certainty of Evidence: moderate. Agreement: 82.3%.**

TAC is a calcineurin inhibitor with immunosuppressive effects similar to those of cyclosporine (CsA). Its mechanism of action involves both T cells immunosuppression and direct antiproteinuric effect due to podocyte cytoskeleton stabilization and reduction of glomerular perfusion pressure through afferent arteriolar constriction [81]. While CsA is associated with a greater risk of dyslipidemia, hypertension, gingival hyperplasia, hypertrichosis and hyperuricemia, TAC is associated with a greater frequency of diabetes and alopecia. Nephrotoxicity can occur with these medications, both acute (TMA, afferent arteriolar vasoconstriction, tubular dysfunction, fluid and electrolyte disturbances) and chronic (glomerular sclerosis, arteriolar thickening, tubular atrophy or interstitial fibrosis) [82].

Studies limited to Asian population, with two RCTs including 402 patients evaluated LN induction therapy with 1000 mg of MMF combined with 4 mg of TAC (divided in two doses) versus CYC NIH. The group with multitarget therapy (MMF + TAC) presented a 33.4% greater rate of CRR (relative risk (RR) 2.37; CI 1.07–5.26; moderate certainty of evidence), with no difference in creatinine levels at 6 months or in the incidence of infections or mortality, compared to CYC. However, these studies excluded patients with creatinine >3 mg/dL; there is a lack of data on long-term histological renal outcomes; and more studies are needed to determine the efficacy and safety of MMF + TAC therapy in other populations [83–85].

TAC for LN is not recommended for patients with creatinine >3 mg/dL, should be avoided for patients with TMA on kidney biopsy, and requires the monitoring of creatinine, blood pressure (BP), and blood glucose after initiation [81–85]. Although there are only a few studies, the association of MMF and CsA can be evaluated when TAC is contraindicated or unavailable [86, 87].

**Practical issues for lupus care:** This consensus also suggests MMF plus TAC as induction therapy, particularly if there is lack of response or impossibility to use CYC or higher doses of MMF (induction dose). However, the limited accessibility of TAC in most regions of Brazil and the scarcity of evidence among non-Asian patients should be emphasized. TAC is not recommended in patients with TMA and/or creatinine >3 mg/dL.

### Question: When belimumab (BEL) combined with standard of care therapy can be indicated for LN patients?

**Recommendation 5: The combination of BEL and MMF can be used as induction therapy according to specific characteristics of patient. Strength of recommendation: conditional. Certainty of evidence: moderate. Agreement: 94.1%.**

A double-blind, randomized, placebo-controlled study evaluated the use of BEL (10 mg/kg intravenously) at 0, 2, and 4 weeks and then every 4 weeks combined with standard therapy (MMF or CYC Euro-Lupus followed by AZA) and reported an 11% increase in renal response rates (PERR—primary efficacy renal response: uPCR ≤ 0.7; GFR ≥ 60 mL/min/1.73 m^2^ or no more than 20% worse than the preflare value; no need for rescue therapy) at 2 years in those who responded to treatment (NNT = 9) and a 10.3% increase in CRR (RR 1.51; CI 1.09–2.12; moderate certainty of evidence), with no difference in the incidence of infections [88]. Post hoc analysis of the pivotal study suggested a better response in subgroups with histological class III or IV, in those with baseline proteinuria < 3 g/g, and in combination with MMF as an immunosuppressant, with twice the chance of achieving CRR as those in combination with CYC Euro-Lupus, which was probably underrepresented in the study compared to MMF. Finally, treatment with BEL combined with standard therapy reduced the chance of new renal flares by 11.6% (*p* = 0.0008) [89].

Thus, BEL must be used in combination with standard therapy (CYC Euro-Lupus or MMF) for LN. Given current studies, BEL can be combined preferably with MMF in patients with class III or IV renal biopsy and proteinuria < 3 g/24 h at baseline. In addition, it should be considered in patients with difficulty in reducing GC dose, high risk of progression to damage, associated extrarenal manifestations, high risk of relapse or frequent relapses, and a high risk of progression to CKD. It should not be recommended to treat LN for those on renal replacement therapy or with a GFR < 30 mL/min, except in cases of extrarenal manifestations [88–91]. The subcutaneous presentation can also be administered at a dose of 400 mg weekly in the first month followed by a dose of 200 mg weekly thereafter.

**Practical issues for lupus care:** BEL can be combined preferably with MMF to enhance renal response, decrease the risk of new flares and help to reduce oral GC dose. It should be considered in patients with difficulty in reducing GC dose, high risk of progression to damage, associated extrarenal manifestations, high risk of relapse or frequent relapses, and high risk of progression to CKD.

### Question: Can TAC be used as immunosuppressant in monotherapy in induction therapy in patients with LN?

**Recommendation 6: TAC as an immunosuppressant in monotherapy can be used as induction therapy if MMF, CYC, MMF + TAC or BEL + MMF cannot be used. Strength of recommendation: conditional. Certainty of evidence: moderate. Agreement: 100%.**

Research conducted exclusively on Asian population suggest that TAC exhibit comparable efficacy to CYC and MMF in LN induction treatment. The dose used ranged from 0.05 to 0.1 mg/kg/day, divided into two daily doses. Tacrolinemia is monitored, with a target of 4 to 8 ng/mL, 6 to 8 ng/mL, or 5 to 10 ng/mL, depending on the study. Patients with severe renal impairment and crescentic glomerulonephritis were excluded from the studies [72, 92–95]. Compared to that of CYC NIH, TAC is noninferior during induction treatment of LN [93]. A 2-year multicenter RCT revealed a similar response rate and increased risk of leukopenia and gastrointestinal symptoms in the CYC NIH group [92]. A small prospective RCT evaluated 60 patients with active LN treated with CYC, MMF, or TAC and reported similar complete renal response (CRR) (30%, 45% and 40%, respectively; *p* > 0.05) and partial renal response (PRR) (60%, 75% and 70%, respectively; *p* > 0.05) with rapid improvement in proteinuria and an increase in serum albumin in the TAC group [72]. According to the analysis of the RCTs included in the consensus, there was a better rate of CRR (RR 1.38; 1.09–1.75) for TAC than for CYC, with a moderate certainty of evidence. MMF and TAC had similar CRRs (RR 0.91; 0.65–1.27). Additional studies involving diverse populations is warranted.

**Practical issues for lupus care:** This consensus suggests that TAC monotherapy can be considered as induction therapy if MMF, CYC, MMF + TAC or BEL + MMF cannot be used. TAC accessibility is limited in most regions of Brazil.

### Question: Can voclosporin be used as an induction treatment for LN?

**Recommendation 7: The combination of MMF and voclosporin may be considered for induction therapy after its approval by Brazilian regulatory agencies. Strength of recommendation: weakly in favor. Certainty of evidence: low. Agreement: 94.1%.**

Voclosporin is a calcineurin inhibitor that is analogous to CsA but has better metabolic stability and a similar mechanism of action, blocking proliferation and responses mediated by T lymphocytes and stabilizing renal podocytes [81, 96, 97].

Phase II (AURA-LV) [96] and a phase III (AURORA 1) [97] studies reached their primary endpoints. AURORA 1 evaluated voclosporin 23.7 mg + mycophenolate 1 g twice daily with low-dose corticosteroids in LN patients with class III and IV with UPCR ≥ 1.5 g/g or class V UPCR ≥ 2 g/g and found a greater chance of achieving the primary endpoint at 52 weeks (41% versus 23%/OR 2.65; 95% CI 1.64–4.27; *p* < 0.0001) (97). Voclosporin was approved for the LN treatment in the USA in 2021. In Brazil, voclosporin has not been approved by regulatory agencies yet, and the posology for this combination (10 pills/day) raises concerns regarding adherence.

**Practical issues for lupus care:** Although studies demonstrated the effectiveness of voclosporin plus MMF as induction therapy for LN, the former drug is not approved in Brazil.

### Question: Can CsA be used as an induction therapy in LN?

**Recommendation 8: CsA as an immunosuppressant in monotherapy is not recommended for induction therapy. Strength of recommendation: strongly against. Certainty evidence: very low. Agreement: 82.3%.**

CYCLOFA-LUNE, an open, multicenter RCT, evaluated the use of CYC or CsA for the induction and maintenance therapy of LN proliferative with preserved renal function and reported similar results between the two drugs, considering response and adverse effects [98]. Due to the limited number of RCTs and the associated risk of drug toxicity, especially in chronic use, panelists do not recommend CsA as immunosuppressant in monotherapy for Class III or IV (± V) LN.

**Practical issues for lupus care:** Due to the limited number of studies, this consensus does not recommend CsA monotherapy for proliferative LN induction therapy. Caution with arterial hypertension, hyperglycemia, hypertrichosis and worsening renal function is important when using CsA.

### Question: Can leflunomide (LFN) be used as an induction therapy for LN?

**Recommendation 9: LFN as immunosuppressant in monotherapy is not recommended for induction therapy. Strength of recommendation: strongly against. Certainty of evidence: very low. Agreement: 88.9%.**

LFN, an inhibitor of dihydroorotate dehydrogenase, has antiproliferative and anti-inflammatory effects by decreasing T cells and B cells. Despite the low quality of available evidence, some observational studies and one small RCT suggested that LFN is safe and well tolerated and may be an effective induction treatment for proliferative LN. These studies predominantly involved Asian SLE patients [99–102].

A recent RCT study in Chinese patients evaluated LFN 40 mg/day for 3 days followed by 20 mg/day versus CYC 0.8–1.0 g monthly as an induction treatment for proliferative LN and reported similar efficacy and safety profiles. The study included fewer patients than the original sample size calculated, and the follow-up only lasted 24 weeks [103]. A systematic review encompassing 254 patients evaluated the efficacy and safety of LFN compared to CYC in Chinese adults with LN and, despite the small sample and high heterogeneity of the studies, suggested a similar favorable safety profile of LFN in these patients [104].

Given the overall low quality of the evidence regarding the use of LFN for proliferative LN induction therapy, coupled with the predominantly Asian population and limited follow-up duration in the available studies, the panel strongly advises against the use of LFN for proliferative LN induction therapy.

**Practical issues for lupus care:** Due to the limited number of studies and quality of evidence, this consensus does not recommend LFN monotherapy for LN induction therapy.

### Question: Should pulse therapy with methylprednisolone be combined with CYC throughout induction therapy for LN?

**Recommendation 10: Monthly glucocorticoid pulse therapy is not recommended during induction therapy. Strength of recommendation: strongly against. Certainty of evidence: very low. Agreement: 88.2%.**

Since the discovery and application of GC in the 1950s, this class of medication has been very important in the treatment of various immune-mediated rheumatic diseases [105]. It has been used as an anchor medication for many years and is combined with standard therapy. On the other hand, it presents a risk of serious adverse events and damage [65].

A randomized study evaluated the effect of combined therapy with CYC NIH and pulse therapy with monthly methylprednisolone (1 g/m^2^ body surface area) versus each therapy alone. There was no difference between combined therapy and CYC alone, with a possibly greater risk of adverse events [106, 107]. Thus, pulse therapy with methylprednisolone combined with CYC or other immunosuppressants throughout the induction treatment is not recommended.

**Practical issues for lupus care:** monthly GC pulse therapy is not recommended, in order to avoid damage accrual.

## Maintenance therapy

### Question: Can MMF or AZA be used as maintenance therapy for LN?

**Recommendation 11: Both MMF or AZA can be used as maintenance therapy. Strength of recommendation: conditional. Certainty evidence: very low. Agreement: 82.3%.**

MMF and AZA are the most indicated medications for LN maintenance treatment [29]. The MAINTAIN study included 105 European patients who received AZA 2 mg/kg/day or MMF 2 g/day after induction treatment with CYC Euro-Lupus [108], and long-term analyses confirmed the lack of superiority of any of the strategies regarding renal activity and progression to CKD [39]. The multicenter ALMS study evaluated 227 patients who received AZA or MMF after induction therapy with CYC or MMF and revealed the superiority of MMF in terms of renal relapse, progression to CKD, and the need for rescue therapy. Importantly, ALMS multiethnic cohort, including Europeans, Asian, Hispanic, and African American populations, demonstrated the superiority of MMF in these populations [109]. Regarding adverse events, there was no difference between the number of infections or malignancies, and those using AZA had more hematological adverse events [110].

We recommend the use of MMF 1–2 g/day or AZA 2 mg/kg/day for LN maintenance treatment. The choice of strategy should consider patient individual characteristics. For those who receive induction treatment with MMF, it is recommended to maintain the same drug during maintenance treatment according to data from the ALMS study which showed that these patients have a worse response when switching to AZA. However, AZA should be used as maintenance therapy in pregnancy and for those planning pregnancies as well as those intolerants to MMF [29, 109]. The availability, cost and dosage regimen (frequency) of the medication should also be taken into consideration [29, 31].

**Practical issues for lupus care:** both MMF or AZA can be used as maintenance therapy. For those who receive induction treatment with MMF, it is recommended to maintain the same drug during maintenance treatment. AZA can be used during pregnancy and pregnancy planning, and has a better posology than MMF, with fewer pills a day and less gastrointestinal intolerance.

### Question: Can calcineurin inhibitors (CsA or TAC) be used as maintenance therapy for LN?

**Recommendation 12: Calcineurin inhibitors (TAC or CsA) can be used as maintenance therapy in patients who cannot use MMF or AZA. Strength of recommendation: weak against. Certainty evidence: very low. Agreement: 94.1% for CsA and 100% for TAC.**

Few quality RCTs have evaluated the outcomes of these drugs in maintenance therapy, so they are not recommended as first-line therapy. A study in a China compared the efficacy of TAC (with a serum concentration of 4 to 6 ng/mL) with that of AZA (2 mg/kg/day) as maintenance therapy for only 6 months and revealed no differences in CRR, PRR or adverse events [111]. An Italian multicenter, randomized, controlled study compared the efficacy of CsA and AZA in LN maintenance treatment after induction therapy with oral CYC for 3 months. Proteinuria reduction occurred in both groups but was more rapidly with CsA. However, the small number of patients and of renal flares in both groups precluded definite conclusions [112].

**Practical issues for lupus care:** Calcineurin inhibitors (TAC or CsA) can be used as maintenance therapy in patients who cannot use MMF or AZA. Awareness of calcineurin inhibitors renal acute and chronic toxicity is necessary.

### Question: Can LFN be used as maintenance therapy for LN?

**Recommendation 13: LFN can be used as maintenance therapy in patients who cannot use MMF or AZA. Strength of recommendation: weak against. Certainty of evidence: very low. Agreement: 94.1%.**

Only one open-label, noninferiority RCT, evaluated LFN and AZA for 36 months maintenance therapy in Chinese patients with proliferative LN, who achieved complete renal response after monthly induction therapy with monthly CYC for 6–9 months. LFN was found to be noninferior to AZA in terms of efficacy and safety [113]. The lack of evidence regarding LFN efficacy in proliferative LN maintenance therapy, coupled with the limited data form a single study involving solely Asian patients, precluded the panel from endorsing LFN as first-line therapy in this scenario. However, in patients who do not respond to MMF or AZA or who present considerable toxicity, LFN may be considered a therapeutic option.

**Practical issues for lupus care:** LEF can be considered for maintenance therapy in patients who cannot use MMF or AZA.

### Question: Should CYC be used in maintenance therapy for LN?

**Recommendation 14: CYC is not recommended for maintenance therapy. Strength of recommendation: strongly against. Certainty of evidence: very low. Agreement: 94.1%.**

Only one open-label RCT after LN induction treatment compared CYC (0.5 to 1.0 g/m^2^ every three months), AZA (1 to 3 g/kg per day), and MMF (0.5 to 3 g/day) for 1 to 3 years as maintenance therapy in a Chinese patient. The composite outcome of patient and renal survival was greater in the AZA (*p* = 0.009) and MMF (*p* = 0.05) groups. LN recurrence was less frequent among those using AZA (*p* = 0.02), with a greater frequency of hospitalizations, amenorrhea and infections with CYC [114]. The CYCLOFA-LUNE, an open, multicenter RCT, evaluated the efficacy of CYC versus CsA for both induction and maintenance of proliferative LN with preserved renal function. The study revealed comparable treatment response rates and adverse effects with both drugs [98].

Although CYC has historically been used as LN maintenance therapy, its adverse effects, especially those related to prolonged exposure time and cumulative dose, prompts caution. With the availability of newer, more effective therapeutic options with a lower risk profile of adverse events, CYC should not be recommended as maintenance therapy.

**Practical issues for lupus care:** CYC is not recommended for maintenance therapy due to adverse events related to CYC prolonged exposure and cumulative dose.

## Treatment of class V LN

**Fig. 4:**
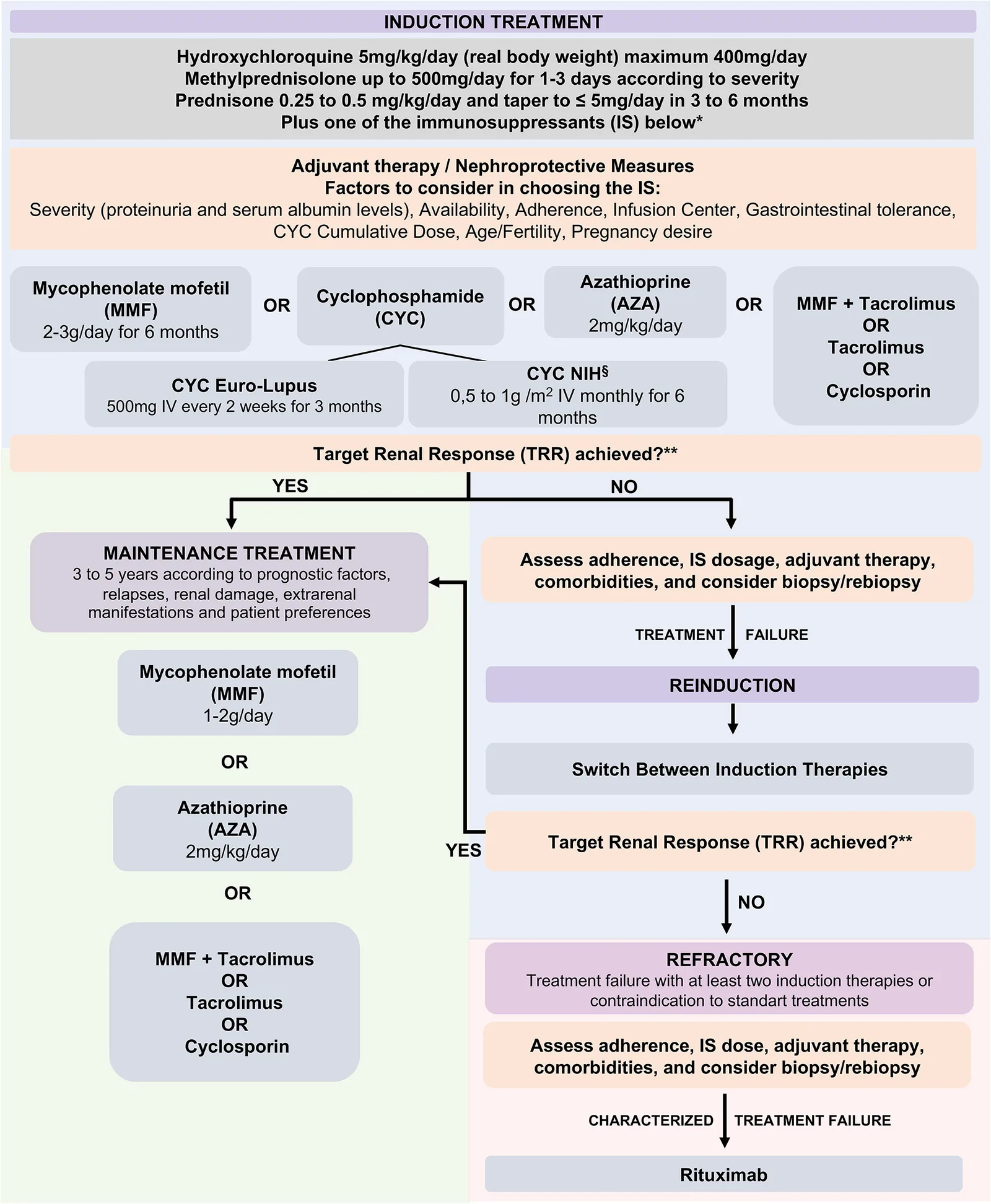
Treatment of class V LN. *Factors to be considered when choosing immunosuppressants: severity (proteinuria and serum albumin levels), availability, adherence, infusion clinic availability, gastrointestinal tolerance, CYC cumulative dose, age/fertility, desire for pregnancy. **Target Renal Response (TRR): reduction in proteinuria by 25% at 3 months, 50% at 6 months, and proteinuria < 0.8 g at 1 year associated with maintenance or improvement (±10% baseline) in renal function. Nephrotic proteinuria at baseline may require another 6–12 months to achieve TRR and in such cases, immediate therapy changes are not necessary if proteinuria is improving. If clinical or laboratory worse within 3 months, therapy changes should be considered. ^**§**^Severe Disease, Poor prognostic factors, Impossibility to MMF or CYF Euro-Lupu

Pure class V comprises approximately 10 to 20% of LN patients and, for this reason, is underrepresented in most RCTs [115]. Up to 30% of patients may progress to CKD within 10 years [115, 116] and treatment involves the use of corticosteroids and immunosuppressants [95, 117–119] (Fig. 4).

Pulse therapy with IV methylprednisolone should be performed at a dosage of up to 500 mg/day for 1–3 days, followed by oral prednisone 0.25 to 0.5 mg/kg/day, with progressive reduction of the dose and a target dose of ≤5 mg/day in 3 to 6 months.

The following immunosuppressants can be used to treat class V LN: MMF, CYC, AZA, combination of MMF and calcineurin inhibitor (TAC or CsA), calcineurin inhibitor (TAC or CsA). The choice of the immunosuppressant should take into consideration: severity (proteinuria and serum albumin levels), patient adherence, availability/access to medication and infusion centers, pregnancy or lactation, risk of infertility, costs, and patient opinion (Table 3).

Nephroprotective measures are extremely important in the management of class V LN. Blood pressure control and use of antiproteinuric drugs are essential to control proteinuria [120]. It is also important to stop smoking, avoid the use of nephrotoxic drugs and have a low salt diet. Nephrotic proteinuria is associated with dyslipidemia and increased thrombotic risk and preventive treatment is indicated. These measures are described below in session 11 (adjunctive measures beyond immunosuppression).

**Practical issues for lupus care:** this consensus recommends the use of GC and immunosuppressants for the treatment of pure class V LN. Nephroprotective measures, blood pressure control and use of antiproteinuric drugs are essential to control proteinuria in pure class V LN.

## Refractory LN

Rituximab (RTX) is an IgG1 anti-CD20 monoclonal antibody that induces B lymphocyte depletion. LUNAR pivotal study evaluated RTX as an add-on therapy (associated with MMF) and found no differences in CRR (normal serum creatinine or <115% of baseline; normal urinary sediment and UPCR < 0.5) and PRR (creatinine <115% of baseline; urine 1 < 50% erythrocytes of baseline and absence of erythrocyte casts and 50% decrease in UPCR, with 24 h proteinuria < 1 g or <3 g if nephrotic) between the groups RTX+MMF and Placebo+MMF [121].

Data from observational studies, open-label trials and systematic reviews [122, 123] support the use of RTX in LN, with beneficial effects and evidence of renal response, especially in refractory patients. Therefore, we recommend RTX for the treatment of refractory Class III, IV or V LN.

**Practical issues for lupus care:** this consensus recommends RTX for refractory LN (Chart 1).

## Adjunctive measures beyond immunosuppression

**Chart 1:**
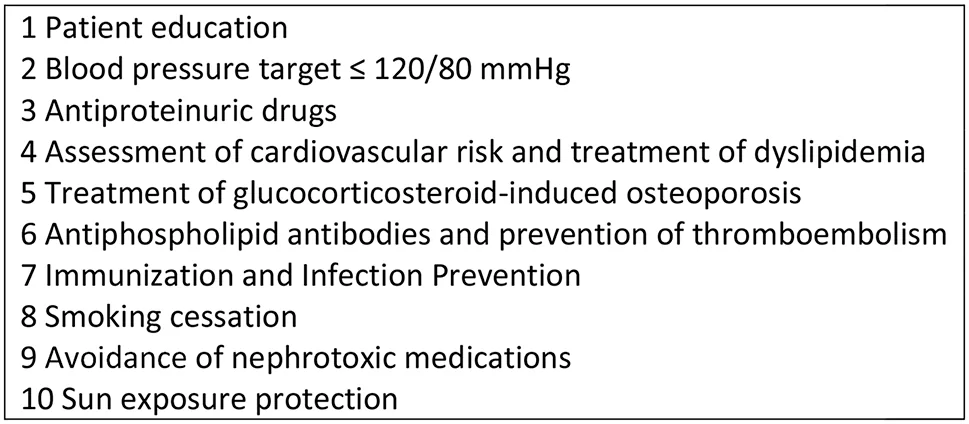
Adjunctive measures beyond immunosuppression in LN patients

### Patient education

Patient participation in the shared decision-making process, including diagnosis, follow up and treatment, can significantly contribute to treatment success. In addition to immunosuppressive treatment, patient must understand the importance of nephroprotective measures and of adhering to their treatment [29, 120, 124]. It is also relevant to raise awareness about photoprotection and smoking cessation, as they are associated with SLE flares [125].

### Blood pressure (BP) target in patients with LN

BP should be controlled at levels ≤120/80 mmHg (degree of agreement 92.3%) with careful consideration for patient tolerance to medications. Recently, KDIGO suggested that, for adult patients with hypertension or non-dialytic CKD, the target of systolic blood pressure should be <120 mmHg. In case of transplant patients, the target was considered <130/80 mmHg [33].

Nonpharmacological measures should include a low-sodium intake diet, moderate-intensity physical activity for at least 150 min per week or at a level compatible with physical and cardiovascular tolerance, maintenance of ideal weight, avoidance of alcohol abuse, and adoption of a cardioprotective diet [33, 126].

### Antiproteinuric drugs

Inhibitors of the renin–angiotensin system (angiotensin-converting enzyme inhibitors [ACEis] or angiotensin receptor blockers [ARBs]) are recommended as first-line therapies for the treatment of patients with hypertension and/or for those with proteinuria even without hypertension, due to antiproteinuric, antihypertensive and nephroprotective effects. These treatments should be discontinued if renal function continues to deteriorate (>30%) and/or if refractory hyperkalemia occurs. The combination of ACEIs and ARBs (double blockade) should not be routinely recommended [33, 126]. To attain the BP target and make medication dose adjustments, home monitoring of BP is recommended.

Inhibitors of sodium–glucose cotransporter 2 (SGLT2) appear to be promising antiproteinuric agents for treating LN and may be useful in patients with CKD (GFR > 25 ml/min) whose proteinuria persists despite immunosuppressive treatment. Data on LN are limited, but there are studies on heart failure and diabetic and nondiabetic nephropathy demonstrating important nephroprotective and cardioprotective effects [127–129].

### Dyslipidemia treatment in patients with LN

SLE should be considered an independent risk factor for atherosclerotic disease [130–132]. Since LN patients are at moderate risk, the LDL target is <100 mg/dL according to the recommendations of the European Society of Cardiology. Patients with CKD (defined by GFR and/or proteinuria), and those with documented atherosclerotic cardiovascular disease (clinically or unequivocal on imaging) should be classified as either at high or very high risk. In such cases, the LDL targets are <70 mg/dl or <55 mg/dL, respectively (associated with ≥50% LDL reduction from baseline) [133].

### Prevention of glucocorticoid-induced osteoporosis (GIO)

SLE patients have a greater risk of osteoporosis. GC use is the main risk factor for bone loss. In addition, the incidence of fractures varies from 30% to 50% among those taking glucocorticoids for more than three months [134]. Patients should be encouraged to address or discontinue associated modifiable risk factors, such as smoking, alcohol consumption, and physical inactivity/sedentarism [134, 135]. GC prescription should be at lowest effective dose and for the shortest possible duration. Bone densitometry and radiography of the thoracic and lumbar spine are important for evaluating the severity of bone mass reduction and the risk or presence of fracture [135].

A diet rich in calcium (1 g/day) or supplemented in cases of an insufficient diet, associated with the maintenance of adequate serum vitamin D levels (>30 ng/mL) is important for bone mineralization and prevention of GIO [134, 135].

The indication for specific drug treatment takes into account age, fracture risk, glucocorticoid dose and gestational desire [134]. Patients at very high risk (use of prednisone or equivalent ≥30 mg/day for >30 days; prior OP fracture; or BMD T-score ≤ −3.5) and patients > 40 years at high risk should be treated (densitometric osteoporosis or high risk FRAX). Treatment can be considered in patients with moderate FRAX risk. Assessment tool for Brazilian population (>40 years) is available at https://abrasso.org.br/calculadora/calculadora/. FRAX should be adjusted for glucocorticoid dose (when GC dose is >7.5 mg/day, the risk for major fractures is multiplied by 1.15; and for hip fractures is multiplied by 1.2) [135].

The drugs available for the treatment of osteoporosis in Brazil are bisphosphonates, denosumab, teriparatide and romosozumab. Premenopausal women should preferably be treated with oral bisphosphonates. Zoledronate and alendronate are contraindicated when the GFR is <35 mL/min, and risedronate and ibandronate are contraindicated when the GFR is <30 mL/min [135].

### Antiphospholipid antibodies and prevention of thromboembolism

The presence of antiphospholipid antibodies (aPLs) including lupus anticoagulant, anticardiolipin IgG and IgM and anti-beta-2-glycoprotein I IgG and IgM should be investigated in all SLE patients. Approximately 30–50% of SLE patients are aPL positive, and about 15% to 30% will develop antiphospholipid syndrome (APS) [136, 137]. Prophylactic treatment with low dose aspirin (75–100 mg/day) is recommended in SLE patients with a high-risk aPL profile, and may be considered in those patients with a low-risk aPL profile. In the case of APS, treatment should follow specific guidelines according to clinical phenotype [138].

Nephrotic proteinuria, especially membranous glomerulonephritis with hypoalbuminemia (<20 g/dL), is associated with a greater risk of thromboembolic events, especially in the first 6 months after the diagnosis. Despite the absence of RCTs, some authors have suggested a benefit of thromboembolic event prophylaxis in these patients [29]. The serum albumin concentration is a strong predictor of thromboembolic events. KDIGO recommends prophylaxis for thrombosis in patients with an albumin concentration < 2.5 g/dL and associated risk factors (proteinuria > 10 g/day, BMI > 35 kg/m^2^, hereditary thrombophilia, aPL, class III or IV heart failure, recent orthopedic or abdominal surgery, prolonged immobilization, pregnancy, malignancy, previous thromboembolic event, GC use) [33]. Lin et al. suggests prophylaxis in patients with an albumin concentration <3.0 g/dL and associated risk factors [139]. The treatments of choice are heparin or vitamin K antagonists, and prophylactic treatment should be continued until there is a significant improvement in proteinuria levels and serum albumin reaches the level of 3.0 g/dL [33]. The risk of bleeding should be assessed before prescribing thrombotic prophylaxis.

### Immunization and infection prevention

SLE patients have a greater risk of infection, which is an important cause of morbidity and mortality [140, 141]. Thus, early and appropriate prevention, detection and treatment are essential in infection management of immunosuppressed patients.

Screening for hepatitis A, hepatitis B, hepatitis C, HIV, and syphilis is recommended before starting immunosuppressive therapy. Prophylactic treatment should be considered in patients with a history of cured hepatitis B (anti-HBc+/anti-HBs+) who in planning B-cell-depleting therapy or intense immunosuppression. In patients with active hepatitis C, antiviral therapy should be considered [140, 142]. The prevention of *Strongyloides stercorallis* hyper infection syndrome with the use of antiparasitic drugs is recommended for patients receiving high doses of glucocorticoids, especially in pulse therapy regimens. Ivermectin (200 µg/kg/day for 2 days, repeated after 2 weeks) is an option with good efficacy and safety profile [143].

SLE patients also have a greater risk of developing tuberculosis than general population. Screening for latent tuberculosis should follow the recommendations of health regulatory agencies in Brazil. Treatment of latent tuberculosis is recommended for patients with a positive epidemiology and/or positive screening test (PPD ≥ 5 mm or IGRA+) and/or radiographic findings suggestive of previous contact with tuberculosis [4, 142].

*Pneumocystis jiroveci* pneumonia (PJP) is an opportunistic lung infection with high morbidity. It can affect immunosuppressed patients and can be prevented with antibiotic prophylaxis. Although some studies have shown a low incidence of PJP in SLE patients (0.04% to 5%), these patients have a high mortality rate (up to 60%). There are still no specific recommendations for prophylactic therapy for SLE, and this topic remains controversial. The use of prednisone >7.5 mg/day, CYC, MMF, rituximab, interstitial pneumonia and LN are risk factors for PJP, while the use of HCQ seems to be a protective factor. Prophylaxis may be considered for patients with a history of PJP and/or risk factors and/or with persistent lymphopenia (<500/mm^3^) [142, 144–146].

Regarding Covid-19 infection, SLE patients may have a high risk of complications, especially when using RTX or high-dose GC. Preventive measures and vaccination against COVID-19 should be recommended for SLE patients, and the use of antivirals should be considered for high-risk infected patients [147, 148] according to recommendations of the Brazilian regulatory agencies.

HPV infection is a risk factor for cervical cancer in SLE patients [149]. Periodic evaluation with oncotic colpo cytology is essential, and vaccination against HPV should be also recommended according to specific guidelines [150].

One of the most effective measures for the prophylaxis of infections is vaccination. Although efficacy may be reduced in SLE patients, most of them develop protective levels of antibodies after vaccination, with a low risk of disease reactivation [142, 151]. Vaccines should be administered, preferably 2 to 4 weeks before the beginning of immunosuppressive/immunobiological therapy or in the period of clinical remission, but vaccine indication should not delay the treatment, especially in severe cases and in those at risk of rapid damage progression. Vaccination against influenza, COVID-19, pneumococcus, meningococcus, *Haemophilus influenzae* B, tetanus, diphtheria, pertussis, hepatitis A and B, HPV, and recombinant herpes zoster is recommended. Vaccines with live attenuated microorganisms are generally contraindicated in immunosuppressed patients. Exceptions include the risk of yellow fever during epidemic situations or for those travelling for endemic areas. In such cases, a shared decision should be discussed with the patients, considering evidence that the vaccine is safe for those with low immunosuppression [152, 153].

Prophylaxis with hyperimmune immunoglobulin is indicated after contact with measles and varicella-zoster immune globulin (VZIG) after contact with chickenpox in the contagious phase. [140, 142].

## Special situations

### Lupus podocytopathy

Lupus podocytopathy is a rare renal manifestation occurring in 1 to 2% of SLE patients and is not included in the classification of LN [25, 26]. Clinically, it presents as nephrotic syndrome resembling class V LN, but light microscopy of the kidney biopsy reveals one of three patterns: normal glomeruli (minimal change type), mesangial glomerulonephritis or focal segmental glomerulosclerosis (FSGS). Thus, podocytopathy should be suspected in patients with nephrotic proteinuria and class I or II LN or FSGS on light microscopy. On immunofluorescence, deposits of immune complexes are absent or restricted to the mesangium (absence of subepithelial or subendothelial immune deposits). The finding of diffuse podocyte effacement (usually greater than 70%) on electron microscopy confirms the diagnosis [154–156].

Treatment is based on observational and retrospective studies and includes the use of glucocorticoids and immunosuppressants (MMF, CsA, TAC, CYC and RTX). In patients with extensive and severe podocyte effacement, calcineurin inhibitors appear to be associated with a higher rate of remission and may be used as first-line therapy [154].

### Involvement of the vascular compartment

Vascular findings secondary to SLE on kidney biopsy include TMA, lupus vasculopathy (with noninflammatory necrotizing lesions with variable immune deposits), and lupus vasculitis (necrotizing and inflammatory vasculitis with infiltration of the transmural vessel wall). TMA is strongly associated with histological chronicity indices; vasculopathy or vasculitis are related to histological activity indices [157, 158].

Clinically, patients with acute TMA may present with arterial hypertension, elevated serum creatinine, microangiopathic hemolytic anemia (presence of schistocytes) and thrombocytopenia. TMA might be suspected in patients with difficult to control arterial hypertension, dysmorphic hematuria and mild or moderate proteinuria (usually <1.5 g/24 h). In case of higher proteinuria, usually glomerulonephritis coexists. Histological findings include wall edema, obliteration or narrowing of the vascular lumen, presence of thrombi in intrarenal vessels of different vascular calibers (acute phase), arterial intimal fibrous hyperplasia, a thyroid-like tubular appearance (pseudo thyroid), an onion appearance, atherosclerosis, arteriolar occlusions and focal cortical atrophy (chronic phase). The prevalence of these findings in the biopsies of patients with LN is 10–39.5% [159–162]. The presence of TMA in renal biopsy is considered an isolated marker of poor prognosis in patients with LN, especially when it is associated with class IV [160–166]. Regarding treatment, there are no RCTs. An observational study suggested the benefits of anticoagulation therapy with warfarin, but the effects on renal outcomes are unclear [167]. In addition to low-dose aspirin, anticoagulation therapy may be considered according to the aPL antibody profile and the risk of adverse events. Treatment of LN and nephroprotective measures (especially ACE inhibitors) are essential. Drugs acting on the mTORR pathway (sirolimus) or on the complement system could represent future treatment options [168, 169]. Calcineurin inhibitors should be avoided in patients with TMA [82]. KDIGO 2024 proposed an specific management fo lupus nephritis and TMA [170].

### Involvement of the tubulointerstitial compartment

Tubulointerstitial disease with or without immune deposits along the tubular basement membrane is a common finding in LN patients. In most patients, it is associated with concomitant glomerular disease, and may be a consequence of glomerular lesions. There is a correlation between glomerular activity and infiltration of interstitial inflammatory cells and between chronic glomerular lesions and tubular atrophy and interstitial fibrosis [171–174]. Interstitial infiltration, tubular atrophy and interstitial fibrosis are independent risk factors for poor prognosis in patients with LN. The severity of tubulointerstitial involvement correlates with the presence of hypertension, baseline serum creatinine, proteinuria, and progressive worsening of renal function. The presence of tubular atrophy and interstitial fibrosis is associated with a twofold increased risk of developing end-stage CKD [172, 173, 175]. It is important to emphasize the risk of tubulointerstitial toxicity caused by CsA and TAC [82].

### Management of chronic kidney disease

Despite treatment, 10% to 30% of patients with LN progress to end-stage CKD, which can be treated with hemodialysis, peritoneal dialysis, or kidney transplantation. Even small elevations in serum creatinine represent significant kidney damage. Besides elevated creatinine, the presence of proteinuria is also associated with worse long-term renal outcomes. In addition to immunosuppressive treatment, patients should receive guidance and nephroprotective and cardioprotective therapies aiming at prolonging renal survival and decreasing cardiovascular risk. Proteinuria reduction is important, as it reflects disease control, and reduces glomerular hypertension, and podocyte damage (probably a major factor in glomerular scarring). Most studies suggest that end-stage renal disease in patients with LN can be largely prevented if proteinuria is reduced to levels below 0.5 g/24 h. Furthermore, progression is slowed if proteinuria is reduced to levels below 1–1.5 g/24 h [33].

### Kidney transplantation

Patients with LN who undergo kidney transplantation have lower mortality than patients with SLE and CKD who remain on renal replacement therapy [176]. The outcomes are similar to those patients who underwent transplantation for other causes of CKD [177]. Thus, kidney transplantation should be considered in patients with end-stage kidney disease as soon as disease activity is controlled [33]. The best long-term results in preemptive transplantation (before the initiation of renal replacement therapy) highlight the importance of early collaboration with the transplant team to facilitate this procedure [178].

Recurrence of LN in the transplanted kidney is uncommon, occurring in up to 10% of cases. The presence of aPL antibodies (including anti-beta2-glycoprotein I IgA antibodies) or APS, is associated with worse transplant outcomes and an increased risk of thrombosis and graft loss [179–181].

### Contraception and management of pregnancy in women with LN

Pregnancies in SLE patients are considered of high risk, with increased maternal and fetal morbidity. Of note, a Brazilian study demonstrated that more than 80% of pregnancies in SLE patients are unplanned, justifying the importance of addressing this topic early with patients and families [182]. Also, pregnancy planning is essential for better maternal an fetal outcomes, and patients with LN should be advised to avoid pregnancy while nephritis is active and for at least 6 months after disease control. Pregnancy should be contraindicated in any of the following conditions: stroke in the last 6 months; pulmonary arterial hypertension; severe restrictive lung disease; CKD classes 3, 4, and 5; heart failure or severe valvular heart disease; and previous episode of severe preeclampsia or HELLP syndrome despite adequate treatment [183–185].

Contraception should be prescribed for patients with pregnancy contraindications, for those with active disease or on teratogenic drugs and for women who do not wish to become pregnant. Available methods include hormonal (progestogen) or cooper intrauterine device (IUD); oral progestogen (desogestrel or drospirenone); intramuscular medroxyprogesterone; or a progestogen contraceptive implant. The use of oral contraceptives containing estrogens can be used in the absence of nephrotic syndrome, if antiphospholipid antibodies are negative, and with low lupus disease activity [185, 186].

Before conception, medication should be reconciled to medications compatible with pregnancy. HCQ should be maintained during pregnancy and lactation, as its discontinuation is associated with a greater risk of maternal–fetal complications [57, 187]. In patients with LN, the immunosupressants AZA, TAC, and CsA are compatible with pregnancy and breastfeeding. GC should be used at the lowest dose necessary to control disease activity [188]. Patients with a history of LN in the last 5 years and receiving maintenance therapy for inactive disease should continue immunosuppressive therapy during pregnancy to prevent LN recurrence. Patients with active LN during pregnancy should also receive immunosuppressive drugs to help control the disease and minimize the use of CEs.

The differential diagnosis between preeclampsia and active LN is challenging, especially when proteinuria and/or arterial hypertension are present. The following factors favor the diagnosis of LN: altered urinary sediment, especially in the presence of dysmorphic hematuria; positive anti-dsDNA; complement consumption; and disease activity in other organs and systems [189]. To support the hypothesis of preeclampsia these factors are relevant: elevated uric acid (>5.5 mg/dL) and an elevated sFLT-1/PLGF ratio [189, 190]. There may be concomitant LN and preeclampsia, and the diagnosis of each condition is important for appropriate treatment since active LN indicates the need for immunosuppression, while preeclampsia requires an efficient BP control and/or to consider the delivery.

The use of low dose aspirin (75–150 mg/day), starting before 16 weeks of pregnancy, is associated with a reduced risk of preeclampsia and preterm birth and is indicated for all SLE pregnant women [191–195]. A calcium rich diet (1 g/day) or calcium supplementation in case of insufficient diet is associated with a 55% reduction in the risk of preeclampsia and its maternal and fetal consequences [196]. Adequate levels of vitamin D are important for maintaining bone mass and preventing osteoporosis.

Prophylactic heparin (enoxaparin 40 mg/day or equivalent) combined with low-dose aspirin is indicated for pregnant women with obstetric APS and may be considered for pregnant women at high risk of thromboembolic events, such as in the presence of high-risk aPL antibodies or in those with active LN and proteinuria >1 g/day [185, 197].

## Discussion

The present consensus aimed to review the main evidence and updates on the treatment of LN, considering the particularities of Brazilian reality. Brazil, a country with continental dimensions, exhibits significant socioeconomic disparities that might be taken into consideration. The analyses performed, including efficacy, safety, values and preferences, costs, equity, acceptability and feasibility, were considered in the decision-making process. Since the last SBR consensus for the treatment of LN in 2015 [4], there were notable advances in both concepts and approaches to the diagnosis and treatment of LN. Besides updating LN treatment, this consensus brings to light Brazilian contributions for the management of LN patients.

Regarding LN diagnosis, kidney biopsy is considered the gold standard, as it allows the evaluation of histological classes and parameters of activity and chronicity, supports differential diagnosis and guides treatment. However, accessibility to kidney biopsy is limited in Brazil and the use of clinical and laboratorial parameters remains the mainstay for diagnosis in most regions of our country. Of note, an instrument developed in Brazil in order to differentiate LN classes was recently published and can help clinical decisions if kidney biopsy is not available [36].

Assessment of proteinuria is essential in the management of LN since early reduction in proteinuria is a predictor of renal response. Two important LN cohorts (Euro-Lupus and MAINTAIN) have shown that 1-year proteinuria level is the best predictor of long-term renal outcome [37, 38]. This finding was confirmed by two Brazilian studies including patients in real life situation with severe disease and distinct histological classes, race, gender and anti-dsDNA profiles [40, 41]. Highlighting the importance of proteinuria in the evaluation of renal response, and in line with international literature, this consensus set the definition of Target Renal Response (TRR), which consists of proteinuria reduction targets at 3, 6 and 12 months, along with preserved renal function. TRR is an easy and effective way to evaluate renal response. The target of proteinuria <0.8 g/day at 12 months was defined according to data from Brazilian patients [40, 41].

Our first recommendation is that HCQ should be prescribed to all SLE patients, except if contraindicated. More sensitive tests, such as OCT-SD, are recommended for detecting early retinal toxicity, but accessibility to them is limited in Brazil. Therefore, avoiding excessive HCQ doses is important to prevent retinal toxicity. Dose of HCQ should be adjusted for real body weight (5 mg/kg/day, maximum 400 mg/day). However, for obese patients (BMI ≥ 30 kg/m^2^), dose of HCQ should be adjusted for ideal body weight (maximum 400 mg/day), as suggested by a recent Brazilian study [59].

Similarly to other international recommendations, this consensus strongly recommends that glucocorticoids should be used at the lowest dose and for the minimal necessary period, in order to prevent damage accrual. Using lower doses of GC, both for induction and maintenance [67, 68], and reaching lower doses in 3 to 6 months is extremely important to limit damage in these patients [65, 66].

For induction treatment, either MMF or CYC Euro-Lupus can be used as first-line treatment, as the main randomized studies showed similar efficacy between the two drugs in controlling LN activity [69, 70, 73–77]. MMF was recently incorporated into the treatment of LN by the Public Health System in Brazil, which simplifies patients’ access to medication. IV CYC is preferred for non-adherent patients to oral medication and CYC NIH should be reserved for patients with more severe forms of LN due to higher CYC cumulative doses and adverse events, including infertility.

Although new therapies have demonstrated benefits in patients with LN in RCTs, their use was considered conditional mainly due to their high cost and difficulty of access in our country. BEL was associated with an 11% greater renal response and with reduced relapse of renal activity [88, 89]. BEL should be considered in patients with difficulty in reducing GC dose, high risk of progression to damage, associated extrarenal manifestations, high risk of relapse or frequent relapses, and high risk of progression to CKD. The use of multitarget therapy (MMF + TAC) has proven to be an alternative to other therapies [83–85]. The association of voclosporin with MMF was also associated with greater renal response, but is not approved in Brazil yet and adherence could be a practical problem since daily dose requires a high number of pills [97].

For maintenance treatment, both MMF and AZA can be used as first-line treatment. MMF is preferred in patients who achieve a good renal response to MMF during the induction phase; however, it is more associated with gastrointestinal intolerance. AZA is easier to administer, generally requires fewer daily pills, is less expensive and is compatible with pregnancy and breastfeeding. The duration of maintenance treatment should be at least of 3 to 5 years. Patients with incomplete response, with multiple previous relapses or with renal damage might need longer periods of immunosuppressive treatment [48].

For pure class V LN, there is scarce literature data since it comprises about 10 to 20% of LN patients [115]. This consensus recommends the use of GC and immunosuppressants for the treatment of class V LN. Nephroprotective measures, blood pressure control and use of antiproteinuric drugs are essential to control proteinuria in class V LN.

Nephroprotective measures should also be emphasized to all LN patients since they are associated with better renal outcome [33]. A Brazilian study showed that a tightly controlled renoprotective protocol is effective in reducing persistent proteinuria in LN [120], which can avoid further unnecessary increases in immunosuppression due to uncontrolled proteinuria. Traditional nephroprotective measures include blood pressure control, renin-angiotensin blockage, low salt diet, smoking cessation, avoidance of nephrotoxic drugs. Despite the scarce evidence in LN, iSGLT2 has been shown to play important nephroprotective and cardioprotective roles in other diseases [127–129], and is also promising in LN.

Comparing with other published guidelines for LN, the II Brazilian Consensus for LN presents some similarities and differences. KDIGO 2024 [170] and Eular 2023 [198] have subtle differences in the definition of renal response target remission, indications of kidney biopsy and strategies of treatment. As mentioned above, in the present consensus, TRR was based in international and Brazilian studies. Regarding treatment, all guidelines reinforced the use of HCQ and low doses of GCs. Both KDIGO 2024 [170] and Eular 2023 [198] considered MMF, CYC, multitarget therapies with BEL + MMF or CYC and MMF + TAC as initial therapy in LN. In the II Brazilian Consensus, economic factors and restricted access to BEL and TAC currently positioned these medications as conditional, taking also into consideration patients characteristics. Another interesting point, KDIGO 2024 [170] and the II Brazilian Consensus emphasizes the treatment of Class V nephritis and adjunctive measures beyond immunosuppression. Altogether, all documents provide an excellent guidance to the growing complexity of LN management and the clinical impact of differences between these guidelines should be analyzed in future studies.

The main limitation of this manuscript is the lack of inclusion of nephrologists and the patients’ perspective on LN treatment, which must be in the agenda in future updates of the guideline and consensus. On the other hand, this consensus has several strengths, including: systematic review and GRADE methodology; rheumatologists with experience in the diagnosis and treatment of LN; decision-making based in important variables (efficacy, safety, values and preferences, costs, equity, acceptability and feasibility) considering the reality of Brazil; discussion of both immunosuppressive treatment and adjunctive measures beyond immunosuppression in all stages of LN; and the proposal of a treatment flowchart for LN.

In the future, there are several additional therapeutics currently being evaluated for the treatment of LN and we expect more patients from Latin America to be included in studies with different ethnicities, studies with pharmacoeconomic analyses, personalized treatments according to biomarkers and histological findings, long-term results with multitarget therapies, and new molecules in phase III/IV studies of LN (obinutuzumab, JAK inhibitors, others).

## Conclusion

This consensus provides evidence-based data to guide LN diagnosis and treatment, supporting the development of public and supplementary health policies in Brazil. However, the autonomy of health professionals must be respected and ensured in relation to the different therapeutic options when based on scientific evidence.

## Electronic supplementary material

Below is the link to the electronic supplementary material.

Supplementary Material 1

## Data Availability

All data generated or analysed during this study are included in this published article [and its supplementary information files].
