# Correction to: II Brazilian Society of Rheumatology consensus for lupus nephritis diagnosis and treatment

**DOI:** 10.1186/s42358-024-00423-6

**Published:** 2024-10-29

**Authors:** Edgard Torres dos Reis-Neto, Luciana Parente Costa Seguro, Emília Inoue Sato, Eduardo Ferreira Borba, Evandro Mendes Klumb, Lilian Tereza Lavras Costallat, Marta Maria das Chagas Medeiros, Eloisa Bonfá, Nafice Costa Araújo, Simone Appenzeller, Ana Carolina de Oliveira e Silva Montandon, Emily Figueiredo Neves Yuki, Roberto Cordeiro de Andrade Teixeira, Rosa Weiss Telles, Danielle Christinne Soares do Egypto, Francinne Machado Ribeiro, Andrese Aline Gasparin, Antonio Silaide de Araujo Junior, Cláudia Lopes Santoro Neiva, Debora Cerqueira Calderaro, Odirlei Andre Monticielo

**Affiliations:** 1 Division of Rheumatology, Department of Medicine, Escola Paulista de Medicina, Universidade Federal de São Paulo (EPM/Unifesp), Otonis Street, 863, 2 Floor, Vila Clementino, São Paulo, SP 04025-002 Brazil; 2 Division of Rheumatology, Hospital das Clínicas, Faculdade de Medicina da Universidade de São Paulo (FMUSP), São Paulo, Brazil; 3 Department of Rheumatology, Hospital Universitário Pedro Ernesto, Universidade do Estado do Rio de Janeiro, Rio de Janeiro, Brazil; 4 Division of Rheumatology, Department of Orthopedics, Rheumatology and Traumatology, Universidade Estadual de Campinas (Unicamp), Campinas, Brazil; 5 Division of Rheumatology, Universidade Federal do Ceará (UFC), Fortaleza, Brazil; 6 Division of Rheumatology, Hospital do Servidor Público Estadual de São Paulo - Instituto de Assistência Médica ao Servidor Público Estadual de São Paulo, São Paulo, Brazil; 7 Division of Rheumatology, Hospital das Clínicas da Universidade Federal de Goiás, Goiânia, Brazil; 8 Division of Rheumatology, Universidade Estadual de Ciências da Saúde de Alagoas, Maceió, Brazil; 9 Division of Rheumatology, Faculdade de Medicina da Universidade Federal de Minas Gerais (UFMG), Belo Horizonte, Brazil; 10 Division of Rheumatology, Department of Internal Medicine, Universidade Federal da Paraíba (UFPB), João Pessoa, Brazil; 11 Division of Rheumatology, Department of Internal Medicine, Hospital de Clínicas de Porto Alegre, Universidade Federal Do Rio Grande Do Sul, Porto Alegre, Brazil; 12 Division of Rheumatology, Santa Casa de Misericórdia de Belo Horizonte, Belo Horizonte, Brazil

**Correction to: Adv Rheumatol 64, 48 (2024)**

10.1186/s42358-024-00386-8

The original publication of this article contained an error in Tables 1 and 2 related to the activity index and the level of agreement. Only the incorrect and correct columns/rows of the table are shown in this correction article, the full tables can be accessed via the original article. The original article has been updated.

Incorrect cells Table 1

**Table:** 

**Activity Index (0–24)** • Endocapillary hypercellularity • Neutrophils and/or karyorrhexis • ***Hyaline deposits (x2)*** • ***Fibrinoid necrosis (x2)*** • ***Cellular/fibrocellular crescents*** • Interstitial inflammation	**Chronicity index (0–12)** • Total glomerular sclerosis • Fibrous crescents • Interstitial fibrosis • Tubular atrophy	**Score** • 0: Absent • 1: <25% • 2: 25–50% • 3: >50%

Correct cells Table 1

**Table:** 

**Activity Index (0–24)** • Endocapillary hypercellularity • Neutrophils and/or karyorrhexis • ***Hyaline deposits*** • ***Fibrinoid necrosis (x2)*** • ***Cellular/fibrocellular crescents (x2)*** • Interstitial inflammation	**Chronicity index (0–12)** • Total glomerular sclerosis • Fibrous crescents • Interstitial fibrosis • Tubular atrophy	**Score** • 0: Absent • 1: <25% • 2: 25–50% • 3: >50%

Incorrect cells Table 2

**Table:** 

Recommendation	Level of agreement
**Induction therapy**	
8. CsA as immunosuppressant in monotherapy is not recommended for induction therapy	***88.2%***
9. LFN as immunosuppressant in monotherapy is not recommended for induction therapy	***82.3%***
10. Monthly glucocorticoid pulse therapy is not recommended during induction therapy	***88.9%***

Correct cells Table 2

**Table:** 

Recommendation	Level of agreement
**Induction therapy**	
8. CsA as immunosuppressant in monotherapy is not recommended for induction therapy	82.3%
9. LFN as immunosuppressant in monotherapy is not recommended for induction therapy	88.9%
10. Monthly glucocorticoid pulse therapy is not recommended during induction therapy	88.2%

