# Supplementary material for: II Brazilian Society of Rheumatology consensus for lupus nephritis diagnosis and treatment
Source: Adv Rheumatol. 2024 Jun 18;64:48. doi: 10.1186/s42358-024-00386-8 (PMC13488729; doi:10.1186/s42358-024-00386-8)
Supplement: Supplementary file 1 [file 42358_2024_386_MOESM1_ESM.docx]

**Supplementary Material 1: PICO Questions**

|  | **Strength of recommendation** | **Certainty of evidence** | **Level of agreement** |
| --- | --- | --- | --- |
| 1. Should hydroxychloroquine (HCQ) be used in every SLE patient with LN? |  |  |  |
| 2. How should corticosteroids be used in induction and maintenance therapy? |  |  |  |
| Induction treatment | | | |
| 3. Is there a preference for mycophenolate mofetil (MMF) or cyclophosphamide (CYC) as induction therapy for LN? |  |  |  |
| 4. Can the combination of MMF and tacrolimus (TAC) be used as an induction therapy for LN? |  |  |  |
| 5. When belimumab (BEL) associated with standard care therapy can be indicated in LN patients? |  |  |  |
| 6. Can TAC be used as immunosuppressant in monotherapy in induction therapy in LN? |  |  |  |
| 7. Can voclosporin be used as an induction treatment for LN? |  |  |  |
| 8. Can CsA be used as an induction therapy in LN? |  |  |  |
| 9. Can leflunomide (LFN) be used as an induction therapy for LN? |  |  |  |
| 10. Should pulse therapy with methylprednisolone be combined with CYC throughout induction therapy for LN? |  |  |  |
| Maintenance treatment | | | |
| 11. Can MMF or AZA be used as maintenance therapy for LN? |  |  |  |
| 12. Can calcineurin inhibitors (CsA or TAC) be used in maintenance therapy for LN? |  |  |  |
| 13. Can LFN be used as maintenance therapy for LN? |  |  |  |
| 14. Should CYC be used in maintenance therapy for LN? |  |  |  |

AZA: azathioprine. BEL: belimumab. CYC: cyclophosphamide. CsA: cyclosporine. LEF: leflunomide. MMF: mycophenolate mofetil. TAC: tacrolimus.

**Supplementary Material 2: Search strategies**

| **Data base** | **Strategy** |
| --- | --- |
| 1. CENTRAL’s search strategy | #1. MeSH descriptor: [Lupus Nephritis] explode all trees  #2. (Lupus Glomerulonephritis) OR (Lupus Nephritis) OR (Lupus Nephritides) OR (Lupus Glomerulonephritides)  #3. #1 AND #2  Filter: Trials |
| 2. *MEDLINE (PubMed) search strategy* | #1. "Lupus Nephritis"[Mesh] OR (Lupus Glomerulonephritis) OR (Lupus Nephritis) OR (Lupus Nephritides) OR (Lupus Glomerulonephritides)  #2. ((clinical[Title/Abstract] AND trial[Title/Abstract]) OR clinical trials as topic[MeSH Terms] OR clinical trial[Publication Type] OR random*[Title/Abstract] OR random allocation[MeSH Terms] OR  therapeutic use[MeSH Subheading])  #3. (animals NOT humans)  #4. #1 AND #2  #5. #4 NOT #3 |
| 3. *Embase (Elsevier) strategy search* | #1. 'lupus erythematosus nephritis'/exp OR 'lupus erythematosus nephritis' OR 'glomerulonephritis, lupoid' OR 'lupoid nephritis' OR 'lupus erythematosus, nephritis' OR 'lupus glomerulonephritis' OR 'lupus kidney' OR 'lupus nephritis' OR 'lupus nephropathy' OR 'nephritis, lupus erythematosus' OR 'nephritis, systemic lupus erythematosus' OR 'systemic lupus erythematosis, nephritis'  #2. 'crossover procedure':de OR 'double-blind procedure':de OR 'randomized controlled trial':de OR 'single-blind procedure':de OR (random* OR factorial* OR crossover* OR cross NEXT/1 over* OR placebo* OR doubl* NEAR/1 blind* OR singl* NEAR/1 blind* OR assign* OR allocat* OR volunteer*):de,ab,ti  #3. #1 AND #2  #4. #3 AND [embase]/lim NOT ([embase]/lim AND [medline]/lim) |
| 4. *Portal Regional BVS Strategy search* | #1. MH:"Lupus Nephritis" OR (Lupus Glomerulonephritis) OR (Lupus Nephritis) OR (Lupus Nephritides) OR (Lupus Glomerulonephritides) OR (Nefrite Lúpica) OR MH:C12.777.419.570.363.680$ OR MH:C13.351.968.419.570.363.680$ OR MH:C17.300.480.680$ OR MH:C20.111.590.560$  Filter: Lilacs |

**Supplementary Material 3: Is there a correlation between protein/creatinine ratio and 24-hour proteinuria in lupus nephritis?**

**Twelve studies r=0,83 (CI 95%; 0,78-0,88). Certain of evidence: low.**
